# Moral contamination: Perceptions of good (but not bad) deeds depend on the ethical history of the actor

**DOI:** 10.3389/fpsyg.2022.1025214

**Published:** 2023-01-19

**Authors:** John Protzko, Jonathan W. Schooler

**Affiliations:** ^1^Department of Psychological Science, Central Connecticut State University, New Britain, CT, United States; ^2^Department of Brain and Biological Science, University of California, Santa Barbara, Santa Barbara, CA, United States

**Keywords:** morality, social cognition, reproducibility, preregistered, moral decision-making

## Abstract

In the majority of moral decision-making research, we are asked to consider the action of someone we know little about—an anonymous actor. This is inconsistent with our everyday judgments of the actions of others. Here we test the novel prediction of whether actions are considered as comparably virtuous or malignant when performed by a good person, an immoral person, or the standard anonymous actor. Across four sets of experiments (nine studies in total), we show that the moral status of the actor contaminates peoples’ evaluations of the virtue of their actions. Even without ulterior motives, people do not judge good acts consistently across actors. We also discover a dose–response relationship where the more immoral the actor has been in the past—the less credit they are given for a good action in the present. This process does not occur for good people performing bad acts, however. Bad acts are bad regardless of who commits them. These results give new insights into the way people evaluate the behaviors of others.

## Introduction

Consider the following hypothetical scenario:

*Thomas volunteers his free time delivering meals to the elderly in need. Is this a morally good thing for Thomas to do?*

People’s answers to the above question, and others like it, can allow us to study how people reason about morality. Typically, when researchers study moral decision-making, nothing else is known about the person performing the act. Does it change the act if Thomas was a war hero? What if he were a serial murderer? Does this background information alter how we view his actions, all else equal? This article addresses these questions. Does the judgment of the act change based only on the moral status of the actor? This is important because when judging the acts of others outside the laboratory, the actor is almost never anonymous and numerous biasing influences could alter the way we interpret behavior and judge acts.

It has been argued that we are able to separate judgments of people from their actions (Uhlmann et al., 2013, 2015). Specifically, “judgments of a person’s underlying moral character can be empirically distinguished from judgments about the rightness or wrongness of an act (as demonstrated by evidence that judgments of acts can be dissociated from judgments of character)” (Uhlmann et al., 2015, p. 72). People think it worse to punch someone in the face than to privately utter a racial slur; yet the racist is a worse person than the striker (Uhlmann et al., 2014).

One study (Siegel et al., 2017) provides initial evidence that people’s assessment of the magnitude of moral infractions can be influenced by more general evaluations of moral character at least when the two are closely aligned. Participants in a dyad had to rate how blameworthy it was for someone else to choose to electrically shock them for money. The less the other person needed to be paid to decide to shock the participant (ostensibly, a marker of their moral character), the worse participants believed each individual act of shocking was. Thus, the more putatively immoral a person was deemed, the worse their individual behaviors were evaluated. Although this study provides preliminary evidence for a relationship between assessments of moral character and evaluations of specific actions, it leaves many questions unanswered. How closely aligned do the actions and the blemishes on one’s moral character need to be for one to affect the other? In the above study, the actions participants evaluated and the dimension on which individuals’ morality was impugned were closely associated. But what if they are disparate as in the *Thomas* example above? Is moral evaluation of people’s specific actions affected by knowledge about their behavior in unrelated domains? If so, how immoral does a person have to be in order for their character to contaminate the evaluation of their actions in another domain? Can knowledge about positive unrelated behaviors mollify negative appraisals, and is the impact of knowing about unrelated positive behaviors comparable to that of knowing about negative ones? In short, there are a host of important questions regarding the moral appraisal of actions that arise once we consider that such judgments can be influenced by more general perceptions of an actor’s moral standing.

Of course, moral evaluation of actions also includes some assessment of the actors’ intentions. If Thomas were volunteering to deliver meals to the homes of the elderly because he wanted to case the houses for the best ones to come back to and rob, we would rightly say his action was not moral.^1^ Similarly, suppose Thomas was running for public office and the only reason he was volunteering was to be photographed giving back to the community to help his voting numbers. These would be cases of tainted altruism (Newman and Cain, 2014) and would likely no longer be viewed as a moral act.^2^ In such circumstances the ‘main effect’ of Thomas’ actions would be a photo opportunity, with the ‘side-effect’ being helping those in need.

When people perform an act that has negative side effects (not the main effects sought after), people believe that those side effects were sought out intentionally; but this intentionality does not emerge for positive side effects (Knobe, 2005; Ngo et al., 2015). If helping those in need were only a positive side effect, we would likely say that Thomas does not intend to help the elderly. Here, however, let us say he is volunteering because family is important to him, he feels bad that these elderly have no family, and that they should be helped. Were this Thomas’s reason for volunteering, we would rightly say his action is still morally good.

Accidentally forgetting to pay a restaurant check is viewed as much less blameworthy than intentionally skipping out on the check (Pizarro et al., 2006); although it is yet unknown how people would judge accidentally walking out on a check if it was done by an immoral, obnoxious individual. Furthermore, this difference in intention arguably changes what the action *is* (e.g., Scanlon and Scanlon, 2009); suggesting results may differ if intention and moral valence of the actor are allowed to differ. Thus, because intentions matter, in all of the studies we investigate here we hold intentions constant, so that participants are judging the same intention-act simply performed by different individuals.

There are reasons to believe other information about the actor, barring intentions, would affect how we reason about the moral status of the act. Halo effects, where someone’s good qualities bleed over into all judgments about them (Nisbett and Wilson, 1977) could operate. If a good person commits an immoral act, halo effects, attributing other good facets (e.g., intelligence, trustworthiness, etc.) to someone with one good attribute (attractiveness), may operate to convince us that the act was not that bad. As an example of the halo effect, made-up poems are considered better if they are attributed to a famous poet (Bar-Hillel et al., 2012). This is not because people are merely claiming the poem is better due to social desirability—they experience the poem in the moment as being superior if they believe a famous author wrote it. Accordingly, just as other things can affect the evaluation of the creative quality of a poem the author has created, so too, in principle the assessment of the moral virtue of an action that someone performs could be influenced by other past acts. Similarly, a reverse halo effect may alter our conceptions of good actions, even if there is no room for ulterior motives or intentions. The closest example is where special education teachers, believing children in a video had oppositional defiance disorder, erroneously attributed unrelated symptoms of ADHD to the children (Abikoff et al., 1993). Thus, bad behavior begat the interpretation of other bad behavior. While speculated about in the popular press (e.g., Glennie, 2011), to our knowledge such reverse halo effects, especially in the moral domain, have yet to be empirically established for cross-modal behavior (e.g., character in one domain altering judgments in an unrelated domain).

An underlying process at work in judgments of the morality of people having an immediate gut reaction that they then reason backward from in an attempt to justify (e.g., Zajonc, 1980). One prominent example is that incest is morally wrong, even if between consenting adults who feel no regrets with multiple forms of birth control (Haidt, 2001). People have an immediate revulsion to such a situation but are unable to justify why it is wrong, often stating ‘I just know that it’s wrong.’ This moral decision-making process begins with a gut reaction that is then rationalized after the fact. It is therefore plausible that a predisposition toward ‘going with your gut’ may drive times where people are unwilling to say a good act committed by a bad actor is still a good act.

Thus, if the moral status of the actor can color our evaluation of their behavior, even when there is no opportunity for bad intentions or ulterior motives, such knowledge can help us understand the descriptive process of act judgments (opposed to the longer tradition of person judgments). Determining whether the moral status of actors can color our judgments of moral and immoral actions is the central purpose of this paper. If we can ignore the moral status of actors, then judgments of moral decision-making would be relatively straightforward. If, however, we bias our judgments of actions in response to the actor, moral decision-making will likely be more difficult than simply looking at an act and judging its morality.

Previous research into behavior-person decision-making has revealed a multitude of possibilities for why people would use the moral status of the actor to influence their judgments of their acts. Early work showed that people generally evaluate a behavior first, then use that evaluation to attribute a disposition to the actor, which then feeds back into the interpretation of the behavior (Reeder and Brewer, 1979). In addition, immoral behaviors are faster to elicit person-attributions, while morally good behaviors may take multiple occurrences before a ‘good’ disposition is attributed to the actor.

The literature on person judgments, which investigates how we judge actors themselves, has a long and equivocal history of research (e.g., Lingle and Ostrom, 1979). People who are told about a person who does moral or immoral things and then are told something inconsistent with the previous impressions alter their beliefs about *a person* more if the inconsistent belief is immoral rather than morally good (Reeder and Coovert, 1986). Furthermore, people are more inspired by actors who used to be bad but then behave in good ways (Klein and O’Brien, 2017). People rate others as more inspiring if they used to do drugs, or had a gambling problem, but no longer do those immoral things. What has yet to be shown, however, is whether people’s perceptions of others affect their evaluations of the acts themselves.

If the moral status of the actor is taken into account when judging behaviors, even holding intentions constant, then our understanding of action judgments needs to recognize we are not simply judging acts. People may reason about the rightness or wrongness of an action not as a function of the act or a calculus of utility or norm/rule compliance, but may indeed engage in reverse reasoning—beginning from an intuition or gut feeling and reasoning backward [as in Haidt (2001)]. Thus, in the following studies, we test this assumption at ever more fine-grained levels, testing the limits of contamination for even minimally moral actions, discovering boundary conditions, and testing whether people’s good moral status alters the evaluation of bad actions.

## Study 1 plus a replication^3^

The purpose of this study was to test moral contamination in a paradigm where no secret ulterior motive could exist and where the basis for characterizing the actor as chronically immoral was unrelated to the good act. We created a story of someone who was extremely immoral (what in our mind was one of the most immoral things a person could do) who performed an act characterized as having been performed spontaneously and without motive.

### Materials and methods

We recruited 68 participants across the United States from an online subject pool. Data collection stopped 1 week after no more subjects were participating. After giving consent, participants were randomly assigned to read the same action performed by a good person, an extremely immoral person, or someone for whom no background information was. Participants in all three conditions were shown a picture of a man and read one of the following stories:

Good Person:

*Pictured here is Leonard. Leonard works for a local organization that is trying to help homeless people get a job, get clean of any substances they are using, and find a place to live. As he is walking down the street he sees a burning building and a child crying that her kitten is still inside. Lenny immediately runs into the building without thinking and completely on impulse, finds the kitten, and brings it to safety.*

Extremely immoral Person:

*Pictured here is Leonard, a man who has raped and assaulted 7 different women. As he is walking down the street he sees a burning building and a child crying that her kitten is still inside. Lenny immediately runs into the building without thinking and completely on impulse, finds the kitten, and brings it to safety.*

Control Person:

*Pictured here is Leonard. As he is walking down the street he sees a burning building and a child crying that her kitten is still inside. Lenny immediately runs into the building without thinking and completely on impulse, finds the kitten, and brings it to safety.*

All participants were asked how moral it was for Leonard to run into the building to save the kitten on a 7-point, unnumbered scale from Not Moral at All to Extremely Moral. On the next page, participants were then asked to explain their reasoning in a couple of sentences. Participants then filled out demographics and were debriefed.

For the replication, we recruited 247 participants across the United States from a different online panel. After giving consent, participants were randomly assigned to read the same action performed by either an extremely immoral person or someone for whom no background information was given (typical of moral research). We dropped the good person condition, as it was consistently indistinguishable from the control in all three previous studies. This suggests firstly that halo effects were not operating for a previously good person performing a good act compared with a control person performing the same good act. In both immoral and control conditions, participants were shown the same picture and read the same text with the following addition: we added that the child was across the street. This was done so participants would not think the little girl was also in the burning building.

### Results and discussion

**FIGURE 1:**
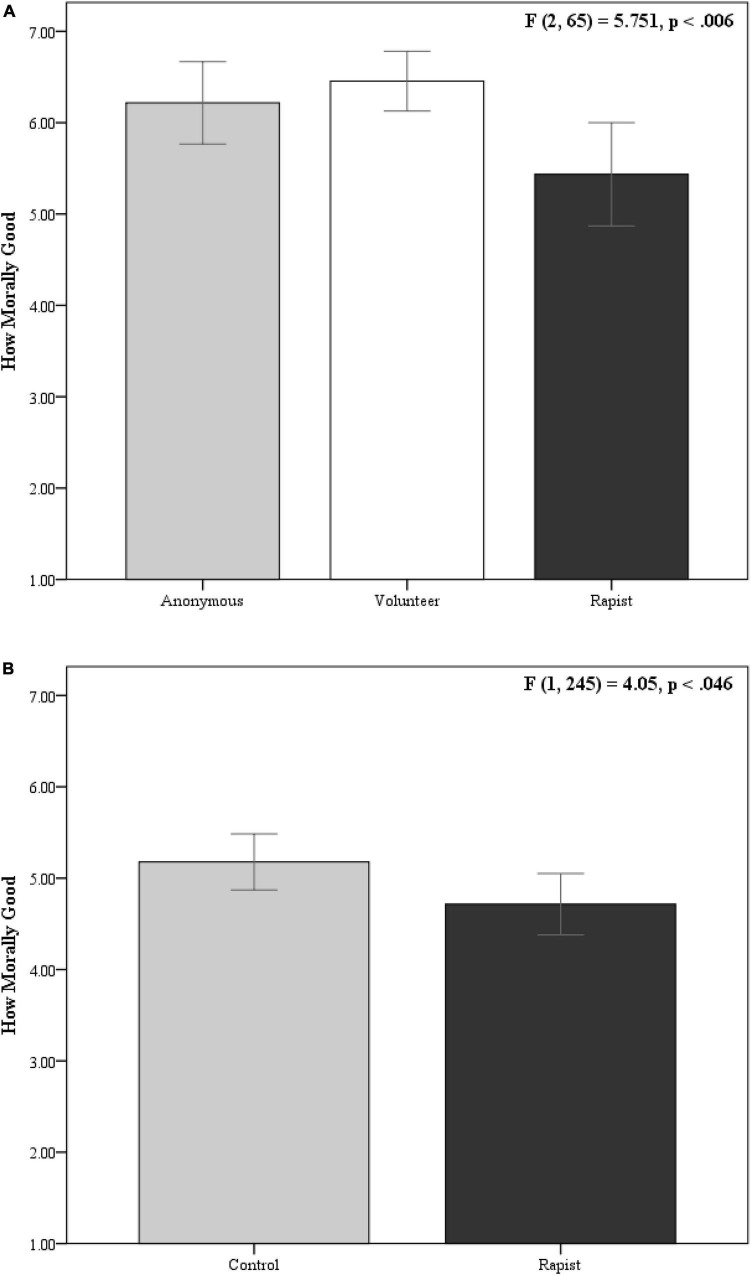
Differences in the moral status of the same action, for the same reasons, performed by different people. Error bars represent 95%CI. **(A)** Shows results from the pilot study; **(B)** shows results from the replication.

There was a significant difference between the three groups [original *F*(2,65) = 5.751, *p* < 0.006; see Figure 1A; replication *F*(1,245) = 4.05, *p* < 0.046; see Figure 1B]. Specifically, people believed it was a very morally good thing for an unknown actor or a volunteer to run into a burning building to save a kitten (original: *M* = 6.217, *SD* = 1.043; *M* = 6.455, *SD* = 0.739, respectively; replication: *M* = 5.177, *SD* = 1.725). It was not nearly as good to run into a burning building to save a kitten if an extremely immoral person did it (original: *M* = 5.435, *SD* = 1.308; replication: *M* = 4.715, *SD* = 1.88). An act committed by an extremely immoral person was considered significantly less moral than if an unknown actor (*M*_*diff*_ = −0.783, 95%CI = −1.551 to −0.015) or a volunteer committed the act (*M*_*diff*_ = −1.02, 95%CI = −1.797 to −0.243; see Figure 1).^4^ An act committed by an extremely immoral person is considered significantly less moral than if a person whose moral background is unknown takes the same act.

While participants still believed the act to be a moral one, it was considered less so than if an extremely immoral person had performed it. The morally good action presented here was performed “without thinking and completely on impulse,” thereby minimizing the possibility of ulterior motives without undermining the virtue of the act itself (as good acts are comparably valued regardless of whether they are deliberate or impulsive; Pizarro D. et al., 2003). Though an exhaustive text analysis of participants’ responses to the open-ended question was not deemed necessary, two judges reviewed the open-ended response for times in which participants commented that the actor intended or meant or wanted to do anything other than save the kitten for the child. No such examples were found. Thus, it appears that participants were not going beyond the instructions to intuit nefarious intentions but did believe that the instructions that the actions were conducted ‘without thought and completely on impulse.’

In this scenario, the action that led the extremely immoral actor to be characterized as such was unrelated to the content of the good act. In addition, there was little room for ulterior motives as the heroic act was committed without thinking. This immediate and without thought description of a heroic act is consistent with real-life accounts of heroism (Rand and Epstein, 2014); which had the advantage of adding realism to this study of decision-making.

## Study 2: Ulterior motives

It is possible that the results from studies one was an artifact of the experimental setup. Participants could be judging the actor and not the act. In the next three studies, we explored possible artifacts in the experimental setup. The three phenomena explored were whether participants believed the immoral actor had ulterior motives, whether participants were answering a different question than the one asked, and whether the presentation of the scenario and response format, affected the results. We did not find any evidence for these artifacts.

The experimental paradigm presents the heroic action as occurring “without thinking and completely on impulse.” This was chosen to not only add validity to the setup but also ensure little room for hidden motives. It was possible, however, that participants did not believe that such a person could do something morally good without such hidden motives. People naturally seek and spontaneously generate explanations for events (Anderson, 1983). Therefore, people may ignore the instructions and generate and explanation that the immoral actor performed the good act for nefarious reasons.

### Methods

To test the possibility of ulterior motives we recruited 314 participants from an online panel to participate in a study about judgments about hypothetical moral situations. Participants were randomly assigned to either the control condition or the extremely immoral actor condition. Participants were asked, “how morally good was it for Leonard to run into the burning building and save the kitten?”. This time, participants answered on a numbered sliding scale from 1 to 7, with labels above 1 (Not morally good at all), 3 (Somewhat morally good), 5 (Morally good), and 7 (Extremely morally good). Responses were recorded to 3 decimal places. Even though previous work has used the phrase ‘how moral’ in the context of asking about good behavior/people (Reeder and Coovert, 1986), we changed the wording here to help make things clearer to participants. Participants were also randomly assigned to answer the following question either before or after determining how morally good the action was: “do you believe Leonard had another motive for running into the burning building?” This allowed us to test whether participants not only fully understood the instructions, but whether they persisted in believing there must be a secret motive.

### Results and discussion

**FIGURE 2:**
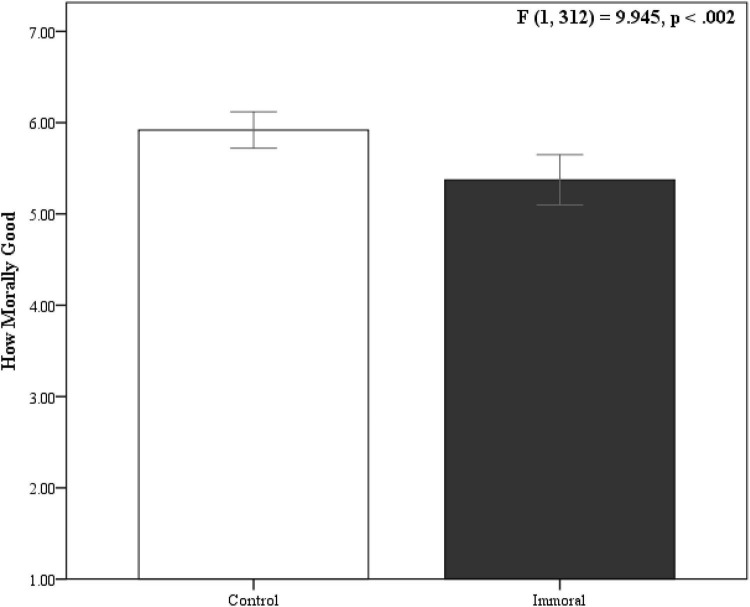
Moral contamination with ulterior motives. Error bars represent 95% CI.

We again replicated the moral contamination effect. People thought it was less morally good for an extremely immoral person to perform a heroic act (*M* = 5.373, *SD* = 1.765) than someone they did not know anything about (*M* = 5.919, *SD* = 1.25; *d* = −0.356, 95%CI = −0.133 to −0.579) (see Figure 2).

In the control condition, 96.9% of participants believed the man had no other motive for running into the building. In the extremely immoral condition, 84.3% believed the man had no other motive. This moral contamination effect, crucially, was not moderated by whether participants thought the bad person had an ulterior motive (*d* = 0.054, *p* > 0.188). In addition, the results remained the same when removing those who thought there was an ulterior motive (*d* = −0.266, 95%CI = −0.0315 to −0.5). The results were also not moderated by order of administration, meaning, or whether participants were asked if the actor had an ulterior motive before or after the question of how morally good it was [*F*(1,310) = 1.734, *p* > 0.18].

These findings strongly support the notion that the moral contamination effect is not simply the product of participants intuiting an ulterior motive despite the framework of the story. The lack of moderation means it does not matter whether participants believe the actor has an ulterior motive. The overwhelming majority of participants in both conditions believed there was no ulterior motive, and believing there was an ulterior motive would not change the results. Although some participants believed, despite instructions, that there *must* be a hidden motive, this did not drive the results. For the most part, participants understood the paradigm, agreed there was no ulterior motive, yet still judged the heroic last less morally good.

## Study 3 – response substitution^5^

In the present paradigm, participants may want to say the *actor* is immoral, but lacking the opportunity to acknowledge this, they may ‘take it out’ on the judgment about the act instead. Answering the question people want to answer is called response substitution (Gal and Rucker, 2011). Participants, for example, will rate the quality of candy lower if they believe the company that made it also markets cigarettes to children. By giving them the opportunity to say the company is immoral, they do not ‘take it out’ on ratings of the candy. An alternate theory of the findings thus far could be participants want to say the actor is immoral and that is the question they are answering.

One way to negate response substitution is to provide participants the opportunity to express their thoughts (see Gal and Rucker, 2011). Accordingly, if response substitution is driving the present findings, if participants are given the opportunity to indicate additional thoughts about the immoral actor there should be no moral contamination effect. To explore whether the findings here were driven by response substitution we randomly assigned 162 participants into either the extremely immoral or control conditions, either with or without the opportunity to give additional thoughts in a 2 × 2 experimental design.

### Methods

To provide participants the opportunity to give additional thoughts, we included the sentence “There is also space below to provide any additional open-ended thoughts or comments you might have” after asking participants how morally good it was to run into the burning building to save a kitten. We chose this procedure to minimize the number of dependent variables taken. Crucially, this manipulation has been shown to eliminate response substitution (Gal and Rucker, 2011, p. 189). Response substitution negation was presented before the collection of the dependent variable. Again, simply giving participants the opportunity to write down additional thoughts, even if they do not take advantage of that opportunity, is the validated method of negating response substitution. If response substitution has been driving the results in the studies until this point, then we would expect a significant interaction of the moral status manipulation and the substitution-negating conditions. In other words, if response substitution is underpinning the present findings then the moral contamination effect should be eliminated/significantly reduced when participants are given the chance to add any additional thoughts.

We also wished to eliminate some other elements from the study that may have been influencing the effect. We removed the picture of the actor and any mention of the picture in the text. We also replaced “How moral was it.” with “How morally good was it…” to eliminate possible confusion.

We recruited 162 participants across the United States from an online panel. After giving consent, participants were randomly assigned to read the same action performed by either an extremely immoral person or someone for whom no background information was given. They were also randomly assigned to receive either the response-substitution-negating instructions or the standard procedure where no chance for alternate responding was given.

### Results and discussion

We again replicated the basic moral contamination finding. Participants who read about a good deed performed by a bad person considered it significantly less morally good (*M* = 5.526, *SD* = 1.569) than those who read about the same action performed by someone they knew nothing about (*M* = 5.987, *SD* = 1.281, *d* = −0.322, 95%CI = −0.64 to −0.004). Crucially, this effect was not moderated by response substitution [*F*(1,150) = 1.807, *p* > 0.181], corresponding to a Bayes Factor of about 2:1 in favor of the null. Meaning, response substitution was not at work in this judgment paradigm.

Therefore, we are confident that response substitution was not driving this result. Across the replications and variants of Study 1, people genuinely believed a good act performed by a bad person is not as good as the same act performed by other people, even when no opportunity for alternate explanations or secret ulterior motives exists. Furthermore, a look at the open-ended responses shows among those who chose to take advantage and add their own open-ended thoughts, no participant believed the actor has an ulterior motive or intention. Those who chose to respond frequently said how the two actions, his past behavior and current actions, are separate and should not and do not affect one another.

We have shown that there is a contamination effect when judging good actions. We do not ignore the actor. In Study 1, we showed that an extremely immoral person who performs a morally good action is given less credit for that act. The behavior is not seen as good as if anyone else had done the act. In studies two and three, we showed this was not a function of the experimental setup, nor an artifact of response substitution, nor were participants intuiting ulterior motives.

## Study 4: Contamination to bad actions

Next, we explored whether the moral contamination occurs in reverse. Namely, do people judge an immoral act differently if performed by a good person? Logically, it could be possible that for all actions, we take the moral status of the actor into account. This could therefore apply to both good and bad acts. A bad act performed by a good person could be seen as less immoral.

The presence of the halo effect (Nisbett and Wilson, 1977), attributing other good facets to someone with one good attribute, may operate to convince people an immoral act was not that bad. Halo effects are often, however, cross-domain, and a different though similar mechanism may operate for people judging bad acts done by good people. Thus, bad acts may be considered not as bad if performed by a good person.

In another vein, however, people do not reason the same way about bad and good events; bad events exert a stronger influence on us than good ones (see Baumeister et al., 2001). When people perform morally good acts, it does not matter whether the act was intentional or accidental; bad actions, however, elicit more blame if they were intentional versus if they were accidental (Pizarro D. et al., 2003). Furthermore, people’s ascriptions of intention change whether the outcome is good or bad. Bad outcomes are more often believed to be intentional than good outcomes (e.g., Knobe, 2003; Nadelhoffer, 2004; Ngo et al., 2015). Although this literature has been criticized for misinterpreting *intentional* with *knowingly* (Guglielmo and Malle, 2010); it is still clear that people interpret the mental states of an actor differently if the outcome is good or bad.

As people reason about good and bad acts differently, especially considering the role of intentions, we examined whether moral contamination effects might show similar asymmetries. It may be, for example, that moral contamination only occurs when actors perform bad acts and not good acts. Thus, unsure about whether moral contamination effects would occur for bad people performing good acts, we tested this possibility in Study 4.

### Materials and methods

We recruited 206 participants from an online sample from around the United States; data collection aimed to stop at 200 participants. Participants were randomly assigned to one of six [3 (immoral, good, and control) × 2 (intentional bad vs. unintentional bad act)] conditions. Participants were asked to consider a hypothetical scenario involving a man named Leonard walking down the street. They either learned the man was: (a) a serial rapist (extremely immoral condition); (b) someone who volunteered at their local homeless shelter (good condition); or (c) were given no background information about the man (control condition). They were told the man was not paying attention and a young child ran headfirst into him. In the intentional condition, participants were told: “[the man], seeing nobody else around and angry at the child, shoves the child to the ground so hard the child breaks their arm.” In the unintentional condition, participants were told: “[the man] was not paying attention and did not see the child running toward him. The child ran into Leonard so hard that the child fell backward and broke their arm.”

All participants were then asked the following two questions: “How immoral was it for Leonard to cause the child to fall and break their arm? There is also space below to include any additional open-ended thoughts or comments you may have” and “How blameworthy is Leonard for the child breaking their arm?” This was done, as before, to control for whether there was response substitution driving any effects.

All answers were on a 1–7 sliding scale with every other point labeled (Not immoral at all), (Somewhat immoral), (Immoral), and (Extremely immoral). Afterward, all participants were then asked the following questions on a separate page: “How good or bad of a person do you think Leonard is? (1–7 Very Good to Very bad scale); and “If a child runs into Leonard in the future, how likely do you think Leonard would be to intentionally push the child to the ground?” (0–100 scale).

### Results

#### Bad person

**FIGURE 3:**
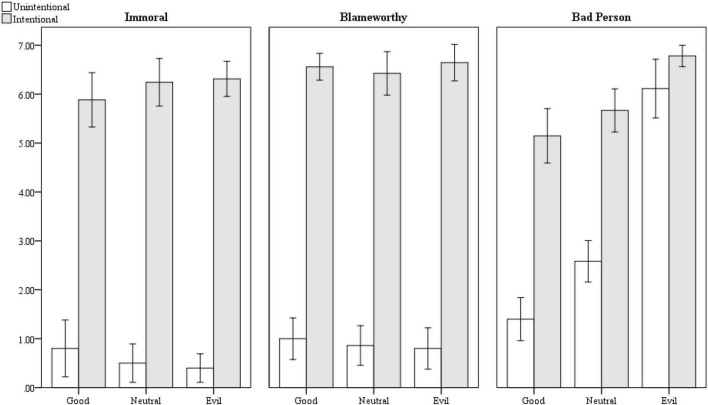
Moral contamination for intentional and unintentional bad actions. Error bars represent 95% confidence intervals.

A significant interaction emerged between intentionality and moral status [*F*(2,200) = 24.103, *p* < 0.001; see Figure 3]; meaning, how bad the person was for intentionally shoving a child to the ground also depended on how immoral they were before shoving the child. *Post hoc* testing using Tukey’s HSD revealed the worst person was the extremely immoral person, regardless of whether they intentionally or unintentionally caused the child to break their arm (no difference between the two; intentional *M* = 6.781, *SD* = 0.608; unintentional *M* = 6.114, *SD* = 1.745). A neutral and good person who intentionally pushed the child were considered equally bad as well (neutral *M* = 5.667, *SD* = 1.242; good *M* = 5.147, *SD* = 1.598), though better than the extremely immoral one who intentionally pushed the child (*p* < 0.01). Finally, a good person who unintentionally ran into a child was considered a better person than a neutral person who did the same (good *M* = 1.444, *SD* = 1.297; neutral = 2.583, *SD* = 1.251; *p* < 0.01).

#### Immorality of the act

Unsurprisingly, participants believed it was much more immoral for someone to intentionally knock a child to the ground and break their arm (*M* = 6.141, *SD* = 1.348) than to unintentionally knock a child to the ground and break their arm (*M* = 0.561, *SD* = 1.275, *d* = 4.258, 95%CI = 3.765–4.752). Crucially, however, there was no effect of how bad the person was, nor an interaction between moral status and intentionality (both *p*s > 0.165).^6^ Thus, we did not observe a halo effect for the act.

#### Blameworthiness

The results were the same for blameworthiness. Participants believed someone who intentionally knocked a child to the ground was much more blameworthy (*M* = 6.641, *SD* = 1.027) than someone who unintentionally knocked a child to the ground (*M* = 0.879, *SD* = 1.211, *d* = 5.025, 95%CI = 4.467–5.583). Again, there was no effect of how bad the person was nor an interaction between moral status and intentionality (both *p*s > 0.728); meaning again there was no halo effect.

#### Likelihood of shoving a child in the future

**FIGURE 4:**
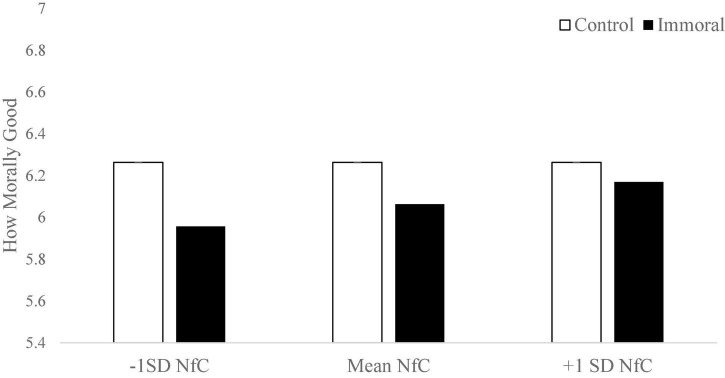
Interaction of need for cognition with moral contamination.

**FIGURE 5:**
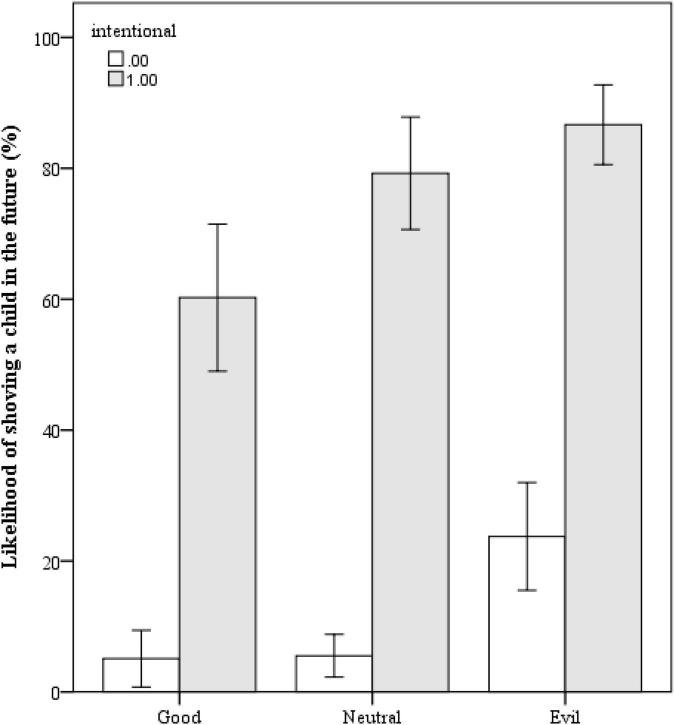
Likelihood of shoving a child who runs into someone again in the future. Error bars are 95% confidence intervals.

The results here are similar to those of judgments of the actor. A significant interaction emerged between intentionality and moral status [*F*(2,200) = 3.334, *p* < 0.038; see Figure 4]; meaning how likely someone who intentionally shoved a child was to do it again depended on their prior moral status. *Post hoc* testing using Tukey’s HSD revealed that a good person who intentionally pushed the child to the ground was considered less likely to do so (*M* = 0.602, *SD* = 0.322) than both a neutral and extremely immoral person who intentionally pushed the child (neutral *M* = 0.792, *SD* = 0.242; extremely immoral *M* = 0.867, *SD* = 0.169; both *p*s < 0.01, no difference between neutral and good). Second, even though it was unintentional, people still believed the extremely immoral person was more likely to intentionally shove a child in the future (*M* = 0.237, *SD* = 0.24) than either a neutral or good person who ran into a child unintentionally (neutral *M* = 0.055, *SD* = 0.097; good *M* = 0.051, *SD* = 0.128, both *p*s < 0.01; see Figure 5).

Though the different dependent variables were highly correlated, they did not hold together as a single factor (RMSEA = 0.272, CFI = 0.957, SRMR = 0.032). This poor model fit was because the item ‘whether the actor was a “bad person”’ did not correlate strongly enough with the other variables to justify a single factor. As the other items were about the act yet the ‘bad person’ item was about the person, it further suggests that for good people and bad acts the two can be separable. Dropping this item from the one-factor model produced superb model fit (RMSEA = 0, CFI = 1, and SRMR = 0). We extracted this single factor using maximum-likelihood estimation and, in this purely exploratory analysis, tested the model again. This factor reflects the immorality of the actor, how blameworthy they are, and how likely they are to shove a child in the future; a factor we shall call *culpability*. As with the reflective DVs, people found the actor more culpable when they intentionally shoved the child rather than unintentionally shoved the child (*d* = 5.616, 95%CI = 5.007–6.225). There was no effect of the moral status of the actor (*d* = 0.04, 95%CI = −0.298 to0.377), nor was there any evidence of an interaction (*d* = 0.153, 95%CI = −0.121 to0.428).

People thought an extremely immoral person was more likely to perform an unrelated immoral act than anybody else. This may be because people think a bad person is a bad person all around. We also found that a previously good person who intentionally broke a child’s arm was considered less likely to do it again than neutral or an immoral person. Presumably because intentionally breaking a child’s arm, while still bad, was considered a ‘one-off’ behavior unrepresentative of the good person. These two findings demonstrate people often think in essential terms about the actors. Bad people are always bad. Good people are always good and if they are not it is unlikely they will ever do it again.

The main purpose of this study was to test moral contamination in reverse. Given the prior findings and the Halo effects, it could well have been expected that the immoral actions of a good person would be seen as less bad or less blameworthy than others. This did not occur. We saw the moral status of the actor altered how people viewed the actor (interaction effects on how bad the actor was and likelihood of behaving badly in the future) but not on judgments of the act (no interactions on blameworthiness and immorality of the act itself.). Thus, the interaction of action judgments and person judgments is asymmetrical.

Although (as seen in Studies 1–3) a bad person performing a good action is not given the same credit as a neutral or good person performing the same action. In this study, a good person performing a bad action was given just as much blame and the act was seen as just as immoral. We saw no evidence for a reverse moral contamination effect. Apparently, judging the morality of good actions involves a different process than judging the immorality of bad actions; different biases and sources are recruited when judging the two. Although contrary to what might have been predicted from research on the Halo effect (Nisbett and Wilson, 1977), these asymmetrical findings are generally consistent with research demonstrating that “bad is stronger than good” (Baumeister et al., 2001), and that the processes associated with judging immoral actions are distinct from those involved in assessing virtuous ones (e.g., Bostyn and Roets, 2016).

Accepting this conclusion of no reverse contamination from the evidence above requires the controversial approach of accepting the null. There are three general approaches to this, and we explore all three here. The first approach is to demonstrate: (i) the null is possible; (ii) the results are consistent with the null; and (iii) that the experiment was a good effort to find an effect (see Frick, 1995). We believe our methods and analyses support all three claims. The second approach to accepting the null is to test two-one-sided tests for whether the observed results are equivalent to the smallest meaningful effect (Equivalency testing, e.g., Schuirmann, 1987; Lakens, 2013). Unfortunately, equivalence testing does not elucidate the problem here. While we conclusively failed to reject the null, there is a lack of data to *definitively* say there is no meaningful effect.^7^ The third way of analyzing the null is through Bayes Factors (e.g., Dienes, 2014). The problem with this approach is that, given minimal sample sizes much smaller than those shown here, any *p* > 0.1 will always be in favor of the null, simply to different degrees. Still, in the interest of completeness, the Bayes Factor for the ‘culpability’ factor is0.07, indicating that the data indeed support the null that there is no reverse moral contamination. Thus, while we cannot be 100% certain that there is no positive moral contamination effect, any evidence for it is lacking here and would indicate such an effect to be, at least, much smaller in magnitude than negative moral contamination.

Study 4 also provides insights into the relationship between people’s prior moral behavior and perceptions of intentions. An extremely immoral person was seen as more likely to intentionally shove a child to the ground than a neutral person, even if they only accidentally ran into the child. This may reflect a lay theory of people where it is assumed bad behaviors in one domain likely reflect a lack of conscience generalizing to other domains. Nevertheless, the same extremely immoral person was not seen as more blameworthy than a neutral person for their action was if it was done unintentionally. A finding that conforms to Pizarro and Tannenbaum (2011) intuition that: “if a person accidentally trips and knocks another in the face with her arm, whether or not she has a criminal record bears little on the assessment of blame because she had little control over the outcome” (Pizarro and Tannenbaum, 2011, p. 99). Apparently, if somebody causes harm we do not alter blame based on their previous moral status, but instead by the intentions of their action.

## Study 5a: The limits of contamination: Setup

Our final set of studies tested the dose–response curve of moral contamination. We tested how immoral a person had to be to contaminate a good action. We also examined how this contamination affects judgments of behaviors ranging from the mundanely good to the heroic. Thus, this investigation allowed us to examine two different questions.

The first question was what the boundary conditions for moral contamination are. In the previous studies, we were able to show that an extremely immoral person contaminated a very morally good act. Would contamination occur for acts that are even more heroic? What about mundane acts like giving spare change to a homeless person? The second question was how immoral does the person have to be to contaminate an act? We previously showed an extremely immoral person could contaminate their good acts. Would the same be true for a less immoral person—for someone who was only mildly immoral?

To test the dose–response relationship, we needed to first gather a range of morally good acts and morally bad acts along a continuum. We required some actions that were mildly morally good to see if moral contamination occurs not only for large feats of heroism but also for small, minor acts. We required some actors who were mildly immoral to see if even minor flaws to a person would contaminate their behaviors. To accomplish this, we constructed a survey where individuals rated how morally good and bad a number of specific actions were. Our goal was to identify a variety of moral actions that were significantly different from one another and represented a range of behaviors from good and bad (see Appendix for all items tested).

### Materials and methods

We generated a list of morally good and bad behaviors along a continuum from the mildest to extreme. We used the lists from Chadwick et al. (2006) in addition to generating our own.^8^ Our final list consisted of 30 good actions and 57 bad actions. We then recruited 187 adults from Amazon Mechanical Turk to rate how good each of the good acts was on a scale from 0 to 100 and how bad each of the bad acts was from 0 to 100. Participants either rated all of the bad items first or all of the good items first. Within each block, the presentation order of acts was randomized.

### Results

We found significant range of moral evaluations for both good and bad acts from the ostensibly mundane to the severe. Both good and bad acts conformed to a linear hierarchy (see Appendix for hierarchy of bad actions).

It is important to note that even the mildest of the morally good behaviors were rated relatively much higher than the mild bad acts. It was difficult to construct a morally good behavior that was mild enough to even fall below the halfway mark.

### Discussion

The purpose of Study 5a was to construct a series of morally good and bad acts to determine the limits and the dose–response relationship of moral contamination. For both good and bad actions, we successfully identified a series of moral behaviors from very mild to extreme that were significantly different from one another to use in the final study.

## Study 5b: The limits of contamination: Execution and dose–response relationships

Drawing on the results of the survey in Study 5a, we selected four behaviors ranging along a continuum from somewhat to extremely morally good. We also selected six behaviors from mildly morally bad to extremely morally bad. These acts were chosen to represent a range of behaviors that showed little to no overlap in terms of judgments of ‘rightness’ or ‘wrongness’ among the 95%CI of previous judgments. In the case of multiple possible acts, we chose ones with the tightest confidence intervals.

The series of bad acts we subsequently used were: talking in the theater, sticking your middle finger up at someone behind their back, selling narcotics to adults, leaving your spouse because they lost their job, and raping multiple women. Each of these was significantly different from one another (all *p*s < 0.05). For the series of good acts, we used: giving a single penny to a homeless person, holding the door open for an elderly woman, chasing down a purse snatcher, and pulling a child out of the way of an oncoming car. This range of good and bad acts allowed us to test the dose–response relationship of moral contamination.

### Materials and methods

We used 1,512 participants from an online sample from around the United States; data collection aimed to stop at 1,500 participants. Participants were randomly assigned to one of 24 (6 × 4) conditions. Participants were asked to consider a hypothetical scenario involving a man named Matt walking down the street. They either learned the man was: (a) someone who talks in the theater (minimally bad); (b) someone who sticks their middle finger up at people behind their back (minor morally bad); (c) a drug dealer who sells to adults (moderately morally bad); (d) someone who left their spouse because that person lost their job (severely morally bad); (e) a serial rapist (extremely morally bad); or (f) they were told nothing about Matt (control condition). All participants were then told that Matt was walking down the street when one of the following occurred: (i) He sees a homeless person up ahead. Without thinking and completely on impulse, Matt reaches into his pocket pulls out all of his spare change (1 penny) and drops it into the homeless man’s cup (barely good); (ii) He sees an elderly woman about to walk into a store. Without thinking and completely on impulse, Matt runs up, opens, and holds open the door for her (somewhat morally good); (iii) He sees someone run past him, steal a woman’s purse, and start to run away. Without thinking and completely on impulse, Matt runs after the purse snatcher and recovers the woman’s purse (morally good); or (iv) He sees a child run out into the street in front of a speeding car. Without thinking and completely on impulse, Matt runs into the street and pulls the child out of the way of the oncoming car (extremely morally good). After each condition, participants were asked how morally good it was for Matt to perform the action. All responses were given on a 1–7 Likert scale from “Not Morally Good at All” to “Extremely Morally Good.” As before, we then included the following: “There is also space below to include any additional open-ended thoughts or comments you may have” to negate any possible response substitution effects.

### Results

Confirming the results from Study 5a, we found average ratings of the good actions were different from one another in the predicted order (all *p*s < 0.003).^9^ See Table 1 for a sampling of reasons people gave for their beliefs. Participants thought donating a penny to a homeless man to be somewhat morally good (*M* = 3.78, *SD* = 1.94). Holding the door open for an elderly woman was considered more morally good (*M* = 5.483, *SD* = 1.49). Chasing down a purse-snatcher was considered morally better than both (*M* = 6.066, *SD* = 1.088). Pushing a child out of the way of an oncoming car was considered the most morally good (*M* = 6.429, *SD* = 0.888).

**TABLE 1:** Reasons participants offered for their judgments.

		Give 1 penny	Hold door for an elderly woman	Chase down a purse snatcher	Pull a child out of the way of a car
Morally good acts					
Moral status of the actor	Control	He wanted to help, so he did what he could	This is what you expect young people to do	He put himself in danger to help an innocent victim of a crime.	If [he] didn’t pull him out of the way the boy would be dead a truly good Samaritan God bless him
	Theater talker	He did more than most.	That is a really nice thing to do	The right instinct	It is good to sacrifice to help society
	Middle finger behind back	… donating a penny shows he made effort (whether poor or wealthy).	We’ve all had those moments of frustration. He was polite where it counted.	It was nice he helped the woman	He has common sense to do the right thing.
	Drug dealer	The thought might be there, but a penny seems insulting	Reflex might be responsible, reflecting upbringing, irrespective of his current moral standing	[He] may be a scumbag, but he also has demonstrated that he has some moral/ethical sensibility	That act of saving the child does not exempt him from being a drug dealer. This tells me he knows better than to deal drugs.
	Spouse abandoner	He is a hypocrite pretending to be pious but not practicing that in his own home with his own wife	Common courtesy. not exactly a moral statement	… does not seem like a person who would leave their spouse for not having a job would be the type to act as a “hero.”	Although he left his wife-[he] still has good intentions in his heart!
	Rapist	That is almost a kick in the gut if you ask me!	[He] is only putting on a show hoping to convince others how good he is, or he only rapes younger women and thinks he is morally fine because he respects his elders. he is one SICK PUP	This type of person will probably keep the purse and definitely not return it or use it as a means to rape the victim	No matter who you are if you save a child from harm it is always morally acceptable thing to do.

**FIGURE 6:**
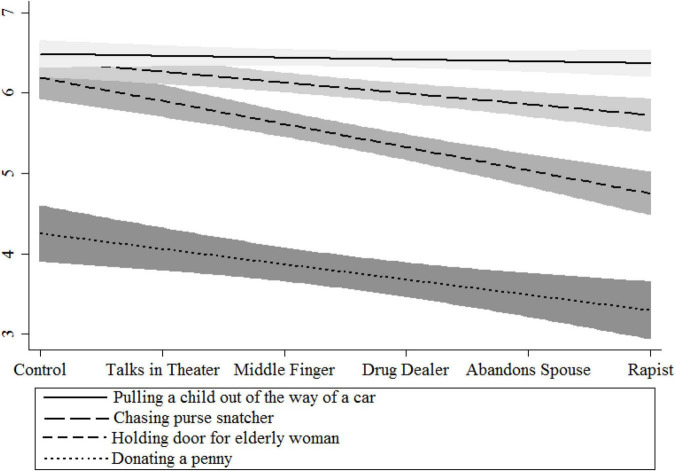
Dose–response fitted lines of moral contamination.

In line with the other studies, there was a significant effect of how bad the person was when judging the morality of their *good* actions [*F*(5,1488) = 16.041, *p* < 0.001]. This was, however, qualified by an act*immoral status interaction [*F*(15,1488) = 3.005, *p* < 0.001]. Follow-up testing was performed using linear contrasts on each action to test dose–response relationships (see Figure 6). Note that this corresponds to statistically predicted judgments in a linear contrast; without such an imposition, as discussed previously, the acts are on average significantly different from each other. Under no condition did the mildest actor (one who talks in the theater) contaminate the actions (all comparisons to controls were equivalent; all *p*s > 0.768).

#### Donating a penny to a homeless man

Participants thought donating a single penny to a homeless man was a morally good thing. Even though it was the least morally good option, participants still rated it higher than the minimum option “not morally good at all” [*t*(375) = 27.8, *p* < 0.001]. A significant linear trend was found for donating a penny to a homeless man; meaning, moral contamination occurred even at the most mundane of good actions. The more immoral the actor, the less morally good giving a penny to a homeless man was [*F*(1,370) = 10.227, *p* < 0.002; $\eta_{\text{p}}^{2}$ = 0.056]. The mildest negative person contaminating regarding the act of giving a penny occurred when the actor was one who sticks their middle finger up behind other people’s backs (*p* < 0.038). Thus, we see even relatively modest negative acts can cause moral contamination.

#### Holding the door open for an elderly lady

We found a linear trend of moral contamination for holding open a door for an elderly woman. Participants found this action to be more morally good than giving a penny to a homeless person. The more extremely immoral the actor, once again, the less good participants thought holding a door open for an elderly woman was [*F*(1,145.758) = 39.184, *p* < 0.001; $\eta_{\text{p}}^{2}$ = 0.148].^10^

#### Chasing down a purse-snatcher

We again found a linear trend of moral contamination for chasing down a purse-snatcher. Participants found this action to be more morally good than holding the door open for an elderly woman. The more extremely immoral the actor, however, the less good participants thought chasing down a purse-snatcher was [*F*(1,164.752) = 15.334, *p* < 0.001; $\eta_{\text{p}}^{2}$ = 0.051].

#### Pushing a child out of the way of an oncoming car

This was by far considered the most morally good behavior out of all of the actions. Interestingly, this is also the only behavior that did not elicit a linear moral contamination effect (*F* < 1). Though the extremely immoral person performing this act was numerically lower than all of the other five groups, this difference was not reliable (*p* > 0.348). It should be noted that there was a strong ceiling effect; all average scores were above 6.244/7. Even taking into account this strong ceiling effect using a tobit regression confirms the numerical difference was not statistically significant (*p* = 0.117). Therefore, we can conclude that moral contamination occurs for all except the most extremely morally good acts.

## Study 6: Evaluating the potential role of axioms of communication and moral intuitionists strategies

A potential concern with the previous studies is the apparent effects of moral contamination could be an artifact of participants’ sensitivity to the axioms of conversation. The way questions and studies are worded gives a lot of information to the participant about what the experimenter may be expecting. Participant’s use of this implicit knowledge about unstated communication inferences that can be drawn from the questions [known as the axioms of communication (e.g., Krosnick et al., 1990; Schwarz, 2014)] are a common threat to the validity of phenomena that emerge through the evaluation of verbal scenarios. People assume, for example, that all information provided to someone, especially if provided by experimenters, is thought by the speaker to be meaningful and should be taken into account. By providing the participant with information about the actor’s underlying character (that they are immoral, for example), participants may infer that the only reason this information is being given is because it is relevant, meaningful, and should therefore be taken into account. If this were the case in the studies presented thus far, it would indicate the phenomenon of finding immoral actor’s behavior less positive could be an artifact of these axioms. Participants may simply be inferring that the experiment wants them to explicitly consider character information, thus guaranteeing our results. To address this, we introduced a new manipulation used to combat the axioms of conversation.

In previous work, for example, people have been shown to have a bias when evaluating probabilities by ignoring base rates (e.g., Kahneman and Tversky, 1973). One reason is that people infer the information being given follows the axioms of conversation. When told a computer randomly selected the information about the individual, people’s judgments do not show such base-rate neglect (Schwarz et al., 1991). Thus, to obviate a similar norm of conversation, that the participant is intuiting important information about the moral status of the actor that the experiments want them to use, we explicitly informed participants the information they learn about the individual before introducing the moral judgment was randomly selected by the computer program.

Furthermore, in Study 6, we evaluated a moral intuitionist (Haidt, 2001) account of our findings by examining the potential role of “need for cognition” in mediating moral contamination effects. Need for cognition represents the tendency and desire to expose oneself to cognitively stimulating events and environments (Epstein et al., 1996). People who are high in need for cognition are considered more reasoned, less likely to make impulsive judgments, and less likely to ‘go with their gut’ (Stanovich, 2011). In an intuitionists-based morality, people have an initial reaction to a scenario and then reason backward to justify it (see Haidt, 2001). If moral intuitionists based processes underpin moral contamination, we would expect the degree to which people ‘go with their gut’ to moderate the results. Specifically, we would predict that those who are more likely to reason based off their intuitions to show greater contamination effects. Therefore, we tested whether the moral contamination effect was stronger for people who were more ‘intuitive’ in their decisions versus more ‘deliberate.’

### Methods

We first administered the Need for Cognition scale to 1,500 participants. These 1,500 participants were drawn from a census-distributed sample of American adults from across the United States (drawn from the online panel CriticalMix). This scale included 18 problems on a 9-point (very strong agreement to very strong disagreement) scale. All questions were presented in random order. These questions included items such as “I like to have the responsibility of handling a situation that requires a lot of thinking”; “I really enjoy a task that involves coming up with new solutions to problems”; and “The notion of thinking abstractly is appealing to me.”

Then, under the cover of a second, unrelated study, participants were randomly assigned to one of four (2 × 2) conditions. The first manipulation either manipulated whether participants were given standard instructions as they had in the previous studies or were in the conversational-negating condition. All participants were told to “Consider a hypothetical person named M—-.” In the conversational-negating condition, we used a manipulation previously shown to negate the axioms of conversation. We told participants: “We are going to tell you some things about M—–. There is a lot to tell, so the computer will randomly select a fact about M—.” As the information they learn about the actor is now ostensibly ‘randomly chosen’, participants no longer feel the need to directly incorporate it into their assessments as it is not considered important to the instructions (as the information is randomly chosen). These procedures have been shown in previous investigations to validly negate certain axioms of conversation (Schwarz et al., 1991; Schwarz, 2014). In the standard instructions condition, no further instructions were given. The second manipulation involved a standard moral contamination manipulation. Participants were randomly assigned to learn that either: “M—- was recently married. When their spouse lost their job, M divorced them and moved to a different state” (contamination condition) or in the control condition: “M— has brown hair.” We chose this level of contamination due to the findings in Study 5b that less extreme immoral actors still contaminate and to balance out the likelihood of still observing a contamination effect to pursue the mechanisms and alternative explanations.

Furthermore, we changed the wording of the dependent variable to “How morally good was it to chase down the purse snatcher?”. Unlike in previous runs, we did this to avoid direct reference to the actor and make the question more clearly about the act.

Therefore, if the effects were driven by conversational norms, we should see an interaction of the contamination and conversational conditions, with moral contamination only occurring in the standard instructions. Furthermore, if the overall effects are moderated by need for cognition, it would help us understand for whom moral contamination was strongest for, and a possible reason for the phenomenon. We constructed a summary score for the Need for Cognition scale and kept all analyses with it as a continuous variable (DeCoster et al., 2009).

### Results

We first tested whether the effect was driven by conversational norms. Overall, we replicated the basic moral contamination effect [*F*(1,1489) = 14.27, *p* < 0.001]; critically, the moral contamination was not affected by whether we negated conversational norms or gave standard instructions (*F* < 1). People who read about a bad person chasing down a purse snatcher thought it was less morally good (*M* = 6.081, *SD* = 1.085) than if they had been a person with no background info given [*M* = 6.277, *SD* = 0.969, *F*(1,1491) = 13.569, *p* < 0.001, *d* = 0.191, 95%CI = 0.089 to0.292]. Therefore, we can confidently say the results of the previous studies were not an artifact of participants ‘intuiting’ that they should explicitly take the moral status of the actor into account via conversational axioms.

Second, we tested whether Need for Cognition moderated the moral contamination effect. The effect of moral contamination was stronger for people who were lower in need for cognition (β = 0.073, *p* < 0.045; see Figure 4). This analysis suggests that moral contamination is an immediate, gut response; while those who are higher in deliberative thought are less susceptible to the effects of contamination.

To probe this interaction, we used a spotlight approach to identify where along the continuum of need for cognition that moral contamination no longer affected people (Hayes, 2013; Spiller et al., 2013). Moral contamination occurred for all participants who were below 0.7 SD above the mean. Thus, we can assume that moral contamination occurs for all but the top 25% people highest in need for cognition. It appears that not only is moral contamination an intuitive process, it is highly prevalent.

### Discussion

Here we begin to isolate *why* moral contamination is occurring. It appears moral contamination occurs because people have an intuitive response when hearing about a bad person performing a good act. Even though they are explicitly told the reasons for this person’s behavior are without ulterior motive, people’s immediate response may be to judge them more harshly. Those who are most susceptible to this intuitive rationalization (low in NfC) are most susceptible. In fact, due to the contamination effect occurring for about 75% of the sample matched to the U.S. adult population, it may be more appropriate to say that it is only the top 25% of those who are not guided by their gut intuitions who are not susceptible to this moral contamination effect.

The absence of a relationship between NfC and moral judgments in the control group (*p* > 0.32) suggests that these participants’ judgments were made independent of processes associated with NfC. It is only when an aspect of the actor comes to light that is inconsistent with their behavior (bad person doing good things) that NfC differences apparently come into play. For all but the top quartile of the population, the inconsistency is too great to be ignored and thus alters judgment about the morality of the behavior itself. This may suggest that NfC is indexing an ability of participants to accept incongruous information and process it separately. This reaction to intuitive demands is consistent with the intuitionist model of moral decision-making. In this model, people have an immediate guttural reaction to a given situation, dilemma, or moral question; they take this as the ‘appropriate’ response and reason backward in an effort to justify it (see Haidt, 2001; Pizarro D. A. et al., 2003). It is only those who are most likely to overrule this quick, intuitive thought process (highest in Need for Cognition) who may be able to hold the discordant view that a bad person can do a good thing for the right reason without any conflict.

## General discussion

Is it morally good to go to war and fight for your country? Is it still morally good if the person is a criminal (see Heighton, 2016)? Are Bill Cosby’s humanitarian work and work to break the racial barriers on television no longer morally good because of his numerous assaults? Is donating your time to volunteer at an elderly home no longer a good act if one is a drug dealer? The results from the studies presented here indeed suggest the actions of an immoral person are contaminated, even without the possibility for ulterior motives and when the action is entirely unrelated to the cause of the actor’s immorality.

The main contributions of this work can be described as follows:

(1) The moral status of the actors contaminates people’s evaluations of good acts; the worse the person, the less credit given for a virtuous act. In studies 1–3, 5b and six, a very good act is seen as less good when performed by an extremely immoral person. This moral contamination occurred when there was no opportunity or possibility for ulterior motives, controlling for possible confounds such as response substitution. It did not matter the format of the questions nor the presence or absence of accompanying photos. It was not an artifact of the axioms of conversation or the experimental setup. Therefore, we are confident the results here represent a genuine mental phenomenon and not an artifact of the experimental paradigm.
(2) Moral contamination of an immoral person on the evaluation of a good act follows a dose–response curve. The more morally bankrupt a person has been in the past, the less credit they are given for the same act. This was true even for the mildest of good acts, such as giving a single penny to a homeless person. The only act immune to such moral contamination was the extreme act of pulling a child out of the way of an oncoming car. This may be because the act was so extremely good nothing could contaminate it; this is expressed in one participant’s comments “No matter who you are if you save a child from harm it is always morally acceptable thing to do.”
(3) Even the most mundane actions are perceived as largely morally good. We saw this in both Studies 5a and 5b. Even giving a single penny to a homeless person was not only considered within the morally good domain, it was rated significantly higher than practically non-moral behaviors.
(4) Even actions performed by mildly immoral people can be contaminated. As shown in Studies 5 and 6, it does not require the worst sorts of people to bring about a moral contamination effect. Acts performed by even modestly immoral people, such as the kind who stick their middle finger up behind someone’s back or leave their spouse for the wrong reason, can be given less credit than when performed by someone we know nothing about.
(5) This moral contamination effect is asymmetric. Although evaluations of good actions are readily reduced by prior immoral behaviors, bad actions remain bad no matter how good the person is who performs them. In Study 4, we found no evidence of a halo effect or reverse moral contamination effect for moral decision-making. People reasoned that a bad act such as intentionally shoving a child was indeed immoral. It did not matter whether the person was good or extremely immoral; the act was still considered immoral and equally blameworthy. The only difference between good and bad actors to emerge was the likelihood of recurrence. A morally good person who performed a bad action was seen as less likely to repeat such actions in the future than neutral or immoral actors.

This work contributes to the growing literature on asymmetry effects in moral decision-making (Pizarro D. A. et al., 2003; Janoff-Bulman et al., 2009; Bostyn and Roets, 2016). Overall, bad actions are bad no matter what, while judgments of good actions take into account the moral status of the actor. This occurs in the absence of any possibility or intuition of ulterior motives on the behalf of the participants.

What causes moral contamination? Contagion accounts (e.g., Rozin et al., 1986) do not adequately account for the data, as such theories are about the transmission of a physical essence [see also Stavrova et al. (2016)]. In addition, disgust, long considered to be a central component of moral decision-making, has been shown not to be causally related to moral decision-making (Landy and Goodwin, 2015).

In the final study, we found moral contamination effects occur for all but those most reliant on reason over intuition. People may have an immediate gut reaction to the idea of bad people performing good acts. Only a minority of these people override that gut reaction with reasoned thought, while the majority of people take the gut reaction as true and work to justify it. As it is widely believed that such intuitive and reasoned processes operate in parallel in moral decision-making (Bucciarelli et al., 2008; Koop, 2013); the appropriate way to understand the findings here is one of relative strength. Those whose reasoned processes hold a greater strength in responses than intuitive processes may successfully dissociate the act from the morality of the individual. This is a minority of the population and as such, moral contamination is likely to be more pervasive.

Furthermore, we did not manipulate intuitionistic mindsets or abilities but simply measured them in Study 6. To more strongly test whether moral contamination effect is automatic, attempts to manipulate intuitionistic strategies and effects through either subtle or overt means should be used in future research to fully understand such process questions.

Any such gut response could be an extension of the fundamental attribution error (Jones and Harris, 1967). Behaviors performed by others are considered to be consistent with a trait of the individual, while explanations of one’s own actions often take into account contextual factors (see Malle, 2006 for meta-analytic evidence for this effect in the context of negative events and an alternate explanation). Since traits may be seen as more permanent than contextual factors, we may believe others are more consistent in their behavior than we are. We could therefore believe the actions of an immoral person are always immoral and discount the morally good actions they may perform for consistency’s sake. There is some evidence that fundamental attribution error-like effects play a role in judging the *immoral* actions of others (Preuss, 2011). People believe an act is less blameworthy when they perform it as opposed to when someone else does. More work, however, needs to be conducted to confirm this as the source of the effects observed here. An attribution error account also does not explain the asymmetry between good and bad acts.

### Relationship to previous literature

This research bears on findings demonstrating the impact of perceptions of people’s moral status on the interpretation of their specific acts (Siegel et al., 2017). Here we see that negative moral status associated with behaviors in one domain can contaminate judgments of the morality of acts in entirely different domains, however. Furthermore, what is needed for moral contamination does not need to be extreme. Even minor impugnments of moral character (i.e., the fact that someone sticks their middle finger up at people behind their back) can undermine the virtuosity of all but the most heroic of acts.

This research shares a superficial similarity with halo effects (Nisbett and Wilson, 1977), although the results operate in reverse. Halo effects demonstrate that someone who is high in some desirable quality is assumed high in other desirable qualities. We saw a hint of this in Study 4 when a good person who intentionally broke a child’s arm was still considered less likely to do it in the future than someone they knew nothing about. The bulk of our findings, however, correspond more to a reverse Halo effect. People believe someone who was immoral was more likely to do immoral acts in the future; when they performed good acts, they were not given the same credit. Furthermore, contrary to the Halo effects we saw no evidence that generally good people were seen as less blameworthy for immoral acts. We found no evidence in Study 4 for halo effects in this domain of moral reasoning.

As negative information is more influential than positive information for judgments about the moral status of an actor (Reeder and Brewer, 1979; Reeder and Coovert, 1986; Skowronski and Carlston, 1987; Wojciszke et al., 1993), it is indeed almost certain our participants accepted the immoral status of the actors in the bad-person-does-good-thing studies.

It could have been possible that a morally good act committed by an extremely immoral person could be seen as *more* moral than if performed by an unknown actor. Accordingly, if a historically bad person engaged in a good act, this might have been seen as a turning point in this immoral person’s life.

In addition, contrast effects (Strack and Mussweiler, 1997) between the information of the actor and the act could have caused an increase in how moral people judged the action. As discussed previously, participants find those who used to be bad but have turned good more inspiring than those who were always good (Klein and O’Brien, 2017). We saw no direct evidence for this redemption story or contrast effects on the *actions* of the actor. Alternatively, despite the fact that participants were instructed to only judge how moral the action was, it may be possible that participants were misunderstanding the instructions, concluding that they should be judging the entire scenario. We think this is an unlikely explanation because in Studies 1–4, participants did not differ on how moral they thought the action was when performed by a good actor compared to an anonymous actor. In addition, it also does not explain the asymmetry of contamination between good and bad acts.

These findings are broadly in line with negativity bias, people seeing bad as stronger than good (Baumeister et al., 2001). Bad actors elicit more contamination than good actors. Bad actions are immoral for their own sake; it does not matter the context. Immoral actors, as well, contaminate what they touch. We demonstrated this was not because people believed the actor had secret ulterior motives. Good actions, however, are not as robust. The people committing them, even if done with no motives, can undermine morally good behaviors.

That the moral status of an actor can change the way people judge their actions, even when all else is held equal, shares some superficial characteristics with previous forms of mental contamination (e.g., Wilson and Brekke, 1994). Mental contamination occurs when either automatic processing or source confusion *bias* thoughts, leading to unwanted conclusions or responses. These intrusions are unwanted because they can lead to non-optimal responses. Moral decision-making, especially in the instances here, does not have necessarily ‘correct’ answers, and the degree to which folk intuitions should be taken into account is debated (e.g., Gert and Gert, 2016). Though most people judge an act to be less moral when performed by an immoral person that does not mean it is *wrong* that an act performed by an immoral person *is not* less moral. Moral differs from mental contamination in that automatic processing intrusion (which Study 6 indicates as a mechanism) creates incorrect or non-optimal biases in mental contamination, but whether these influences are *incorrect* is not decisive in the moral domain. Thus, we believe moral contamination may be a subtype or form of overall mental contamination. The extent to which the two share the same space is a function of how much one can be ‘wrong’ in their moral judgments.

Overall, we see the mechanism of moral contamination is likely an intuitive response to the dissociation of bad people and good actions. People who perform immoral acts are considered immoral as a trait. Seeing that person perform a morally good act goes against our intuition the person is always bad. This inconsistency leads us to discount the act, even if it was done for all of the right reasons.

Only those who are highest in Need for Cognition, those who enjoy paradoxes and difficult problems and being reflective, may be able to hold the inconsistent beliefs of bad people doing bad things without discounting the act. This suggests moral contamination is indeed reflective of an intuitionist morality, reasoning backward from intuitions and gut feelings. Those highest in need for cognition not showing moral contamination is equally important. This suggests that intuitionist morality is not the way people approach moral decision-making unconditionally (cf. Haidt, 2001); at least some people are able to override this habit, though it is uncommon to do so.

An interesting future hypothesis from these findings is that people who have impaired emotional functioning would fail to show moral contamination effects. Patients with focal damage to the ventromedial prefrontal cortex have dampened emotional processing, which also affects their moral decision-making (Koenigs et al., 2007). Specifically, such patients are less affected by emotional responses to complex moral problems, more often choosing the utilitarian option. A possible extension from the present work would be the reduced affective responses of such patients may lead them to be unaffected by moral contamination.

Theories of how people reason about the morality of an action should now take into account more than the intentions of the actor and the outcomes of the act. We can see the moral status of the actor can contaminate judgments about the morality of their actions for most people. An extremely immoral person is not given the same credit for performing a morally good act. Even mildly unethical actors contaminate even mild actions. While this research does not invalidate the entire theory of separation of judgments of people from their actions (e.g., Schweinsberg et al., 2016) it does introduce new complications into the interpretation. In fact, we corroborate the finding that the moral standing of the person does not matter for judgments of their *bad* actions. Our work here shows, however, the asymmetry between judgments of good and bad acts appears in person-centered morality.

Studies such as these do not tell us what *is* good and bad, only people’s intuitions about the morality of actions. Ideally, any account of morality should be able to take into account how people think. If this is the case, we must now understand that people’s moral evaluations of behaviors are colored by more than just what the action is and what the consequences are. Who performs the act, on top of motives and intentions, also guides our judgments about the virtue of an action. This happens asymmetrically, however. We are much firmer about our convictions on what a bad act is than we are about what a good act is. A good act can be marginalized by being performed by an unsavory person.

## Data availability statement

The original contributions presented in this study are included in the article/supplementary material, further inquiries can be directed to the corresponding author.

## Ethics statement

The studies involving human participants were reviewed and approved by the Office of Research Application for the use of Human Subjects; University of California, Santa Barbara. Written informed consent for participation was not required for this study in accordance with the national legislation and the institutional requirements.

## Author contributions

JP and JS conceptualized the studies, wrote the manuscript, and provided the edits. JP programmed the studies and ran the analyses. Both authors contributed to the article and approved the submitted version.

## Figures and Tables

**APPENDIX FIGURE 1:**
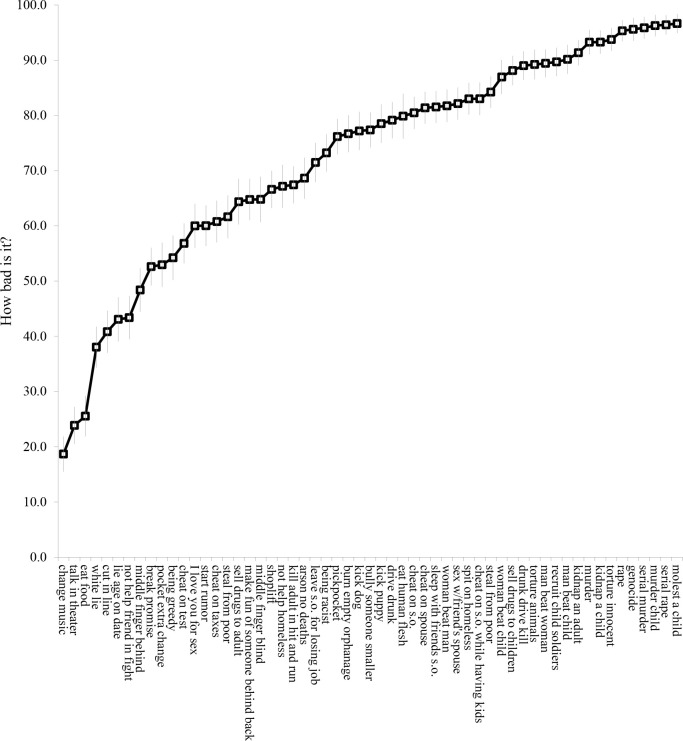
Hierarchy of bad behaviors with 95% CI.

**APPENDIX FIGURE 2:**
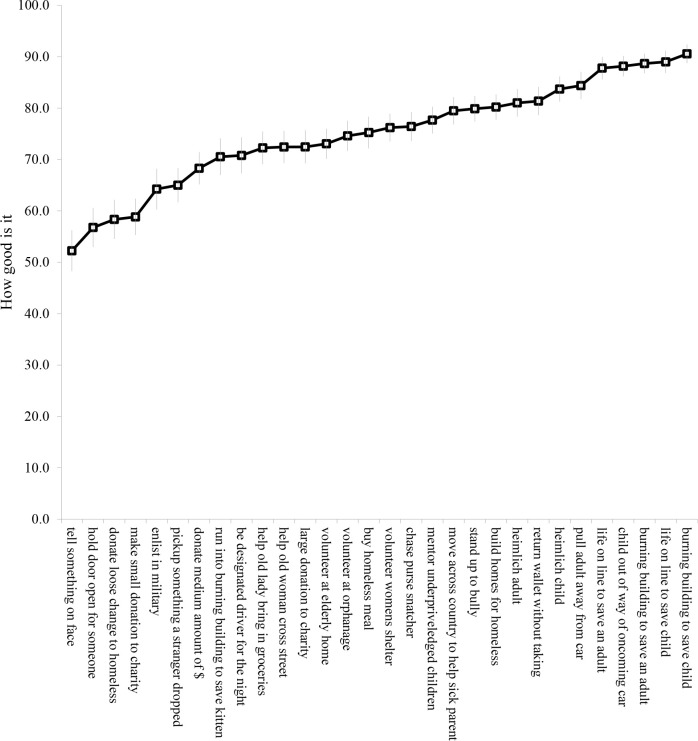
Hierarchy of good behaviors with 95% CI.

## Footnotes

1 Some versions of strict consequentialism would require a calculation on this point, it would be possible that bringing meals provides more good than the ill caused by robbing the house, suggesting even in this situation it would still count as a morally good act. We do not discuss this point further.

2 But again, see note 1 regarding calculation.

3 All pictures, methods, materials, and de-identified data used in this paper were pre-registered before data collection at https://osf.io/dbw67/. For all studies, we report all conditions and variables. We do not exclude any outliers. In cases where participants fail an attention check, we test whether this moderates the effect. If it does not, we include all participants. If it does, we report that in the results section.

4 Study 2 *post hoc* testing done using Bonferroni corrected *t*-tests.

5 In the interest of openness, we report a failure to replicate as well. We conducted a replication of the response substitution study, randomly assigning 206 participants to the same 4 conditions (bad/control and extra space present absent). The overall 2 × 2 ANOVA (bad vs. control/additional space present vs. absent) failed to reach statistical significance (*p* > 0.418). We also failed to replicate the original moral contamination finding (*F* < 1; both with the response substitution variable present or absent from the analysis). Therefore, we reject the notion that anybody can find an effect with a properly powered study. Sampling error can occur at any *N*.

6 *Post hoc* power analysis shows we had 99.93% to detect a difference between the groups given the amount of variance accounted for from analyses in Study 1 (assuming homogenous symmetrical effects).

7 This was tested against both the smallest effect we observed in moral contamination (*d* = −0.322) and smaller effects (e.g., <0.2). We could not reject that the upper bound of our observed *d* = 0.04 was so small as to be meaningless.

8 It was unknown to us at the time, but a series of short vignettes may also be found in Knutson et al. (2010).

9 Results do not change whether Bonferroni correcting or when using Dunnett’s test taking into account heterogeneity of variance.

10 These linear contrasts were adjusted to account for heterogeneity of variance. The results were the same as assuming homogeneity.

## Appendix

For bad acts, participants rated how morally bad the actions were in the following order (note the order does not mean each is statistically worse than the next; see Appendix Figure 1): changing music in a friend’s car without asking, talking in theater, eating food off someone’s plate without asking, telling a white lie, cutting in line, lying about your age on date, not helping a friend in a fight, giving someone the middle finger behind their back, breaking a promise, pocketing extra change after buying something, being greedy, cheating on a test, saying ‘I love you’ just for sex, starting a rumor about someone, cheating on your taxes, stealing from the rich, selling narcotics to adults, making fun of someone behind their back, giving the middle finger to someone who is blind, shoplifting, not helping the homeless, killing an adult in a hit and run, committing arson with no subsequent deaths, leaving your significant other because they lost their job, being racist, pickpocketing, burning down an empty orphanage, kicking a dog, bullying someone smaller than you, kicking a puppy, driving drunk, eating human flesh, cheating on your significant other, cheating on your spouse, sleeping with your friend’s significant other, a woman committing domestic violence against a man, having sex with your friend’s spouse, spitting on someone who is homeless, cheating on your significant other when you have children together, stealing from the poor, a woman beating a child, selling narcotics to children, killing someone in a drunk driving accident, torturing animals, a man committing domestic violence against a woman, recruiting child soldiers, man beating a child, kidnapping an adult, committing murder, kidnapping a child, torturing an innocent person, rape, committing genocide, serial murder, murdering a child, serial rape, molesting a child.

For good acts, participants rated how morally good the actions were in the following order (note the order does not mean each is statistically worse than the next; see Appendix Figure 2): telling someone they have something on their face, holding a door open for someone, donating loose change to someone who is homeless, making a small donation to charity, enlisting in the military, picking up something a stranger dropped, donating a medium amount of money to charity, running into a burning building to save a kitten, volunteering to be the designated driver for the night, helping an old lady bring in her groceries, helping an old woman cross the street, making a large donation to charity, volunteering at an elderly home, volunteering at an orphanage, buying someone who is homeless a meal, volunteering at a battered women’s shelter, chasing down a purse snatcher, mentoring underprivileged children, moving across the country to help a sick parent, standing up to a bully for someone else, building homes for the homeless, performing the Heimlich maneuver on a choking adult, returning a lost wallet without taking anything, performing the Heimlich maneuver on a choking child, pulling an adult away from the way of an oncoming car, putting your life on the line to save an adult, pulling a child out of the way of an oncoming car, running into a burning building to save an adult, putting your life on the line to save a child, running into a burning building to save a child.
